# Physico-chemical analysis of eight pre-mixed hydraulic silicate sealers

**DOI:** 10.3389/fdmed.2026.1851537

**Published:** 2026-06-09

**Authors:** Vasileios Kapralos, Matthias Zehnder

**Affiliations:** Clinic for Conservative and Preventive Dentistry, Center for Dental Medicine, University of Zurich, Zurich, Switzerland

**Keywords:** BC Universal Sealer, Bio-C SEALER ION+, BioRoot Flow, C-Root SP, KometBioSeal, NeoSEALER Flo, One-Fil, TotalFill BC Sealer

## Abstract

**Introduction:**

Pre-mixed root canal sealers containing hydraulic silicate cements are marketed under the “bioceramic” umbrella term. Eight such sealers were investigated to get a comprehensive overview.

**Materials and methods:**

Disclosure of composition was assessed in safety data sheets and instructions for use. Elemental analysis was conducted using energy-dispersive x-ray spectroscopy (EDS). Flowability, radiopacity, and setting time were tested according to ISO norm 6876 (2012). Knoop hardness was tested after 7 d. Samples were immersed in phosphate-buffered saline for up to 28 d and assessed for continued pH induction and release of calcium ions (Ca), which was measured using atomic absorption spectroscopy (AAS). Cumulative aluminum (Al) leaching after 28 d was quantified using AAS.

**Results:**

Dispersing agents were not disclosed in 5 of the 8 sealers, and cement components not specified in 4. As assessed by EDS, 4 of the sealers under investigation were Portland cement-related, and 4 were Al-free silicates. The ISO-requested flowability and radiopacity were met by all the sealers. Radiopacity ranged from 3.4 ± 0.1 to 7.4 ± 0.3 mm Al. Setting time ranged from 122 ± 4 min to 4,258 ± 14 min. The sealers containing Al tended to set quicker and reached a considerably higher 7-d hardness than their Al-free counterparts. All sealers maintained an alkaline pH induction over 28 d, but differed in Ca release. Al leaching was observed with 2 of the 4 Portland cement-related sealers, but to a different degree.

**Conclusion:**

The materials under investigation differed considerably in their basic properties and their propensity to leach elements.

## 1 Introduction

Hydraulic calcium (Ca) silicate cements have a successful history in endodontics. The first material that came to market was ProRoot MTA (Dentsply Sirona Endodontics, Ballaigues, Switzerland), which was mainly Portland cement with bismuth oxide as a radiopacifier (1). MTA was first introduced as a root-end filling material. Subsequently, its clinical applications were expanded to perforation repair and pulp capping (2). Similar to bioactive glass 45S5, the bioactive and antimicrobial effects of Portland cement are related to its induction of a high pH environment and its ability to release ions (3, 4). However, in contrast to bioactive glass, this cement provides a physical barrier after setting that is useful for many dental applications. Portland cement is a natural product, which consists mainly of tricalcium silicate, dicalcium silicate, and tricalcium aluminate. It is derived from limestone (CaCO_3_) and clay, which is the source of the aluminum. To increase material purity, and perhaps also to avoid patent issues, manufacturers have introduced materials that were based on pure synthetic calcium silicate, with or without extra cement components (5, 6). Moreover, the bismuth oxide from the original MTA was replaced by other radiopacifiers with less propensity to discolor dentin (7). This was also when the term “bioceramic” came into use to discern these newer materials from the MTA that contained aluminum and bismuth. Further developments included the addition of calcium aluminates to synthetic CaSi materials to shorten their setting time, improve their mechanical properties, and elevate their acid resistance (8–11).

Based on the clinical success story and the good histological results obtained with the materials described above, the first hydraulic CaSi cement-based root canal sealer was launched in 2007 (iRoot SP, Innovative Bioceramix Inc., Burnaby, BC, Canada). This sealer came in a pre-mixed form in a single syringe, which made it easy to use for dentists, yet retained an element of clinical uncertainty, as it needs water from an external source to set (12). Many more products followed (6). They all claim bioactivity and easy application via a syringe with an attached disposable tip. However, whether these materials should be viewed as similar is questionable. A recent study comparing the physico-chemical properties of 3 premixed single-syringe hydraulic cement–based sealers found considerable variation in their characteristics (13). Interestingly, all sealers maintained some solubility within the test period of 24 h, which goes against the original claim that materials used for the hermetic seal of root canal systems should be inert (14), although transfer of *in vitro* solubility behavior to the actual clinical behavior may not be possible for bioactive sealers (15). Moreover, one sealer released some Al, as was assessed using inductively coupled plasma optical emission spectrometry (13). From a consumer perspective, it is an inherent problem that the manufacturers do not always list their full contents in the respective safety data sheet (16).

The aim of the current study was to scrutinize eight currently marketed pre-mixed hydraulic silicate root canal sealers, all of them marketed as “bioceramics”. First, it was investigated which components were disclosed by the manufacturers and which were not. Elemental composition of the sealers under scrutiny was then studied using scanning electron microscopy coupled with energy-dispersive x-ray spectroscopy (SEM-EDS). The materials were thus grouped into Portland cement-related sealers and aluminum-free counterparts. Material properties were tested according to ISO 6876:2012, including setting time, radiopacity, and flowability, while surface microhardness was assessed after 7 d of setting. The chemical leaching profile was characterized by assessing pH induction and calcium release over time, and by quantifying cumulative aluminum release in Portland cement-related sealers.

## 2 Materials and methods

### 2.1 Materials and their disclosed composition

The sealers under investigation were: BioRoot Flow (Septodont, Saint-Maur-des-Fossés, France), NeoSEALER Flo (Avalon Biomed, Houston, TX, USA), TotalFill BC Sealer (Innovative BioCeramix, Burnaby, BC, Canada), KometBioSeal (Meta Biomed, Cheongju-si, Chungcheongbuk-do, Republic of Korea), One-Fil (MEDICLUS, Heungdeok-Gu, Gongdan-Ro, Republic of Korea), C-Root SP (C-Root Dental Medical, Beijing, China), BC Universal Sealer (Vericom, Chuncheon-si, Gangwon-Do, Republic of Korea), Bio-C SEALER ION + (Angelus, Londrina, PR, Brazil).

Instructions for use were consulted, and safety data sheets were downloaded from the internet to assess material composition as disclosed by the manufacturers and/or distributors.

### 2.2 Sample preparation

Three-dimensional (3D) printed polyethylene terephthalate (PET) cylindrical molds (10 mm in diameter and 2 mm in height) were fabricated using a 3D printer (X1 Carbon 3D printer, Bambu Lab, Shenzhen, People's Republic of China) and used to prepare the sealer specimens for the assessment of setting time, macroscopic and microscopic inspection, elemental analysis, microhardness testing, and assessment of leachates. The sealers were placed into the cylindrical molds designed with enclosed bottom and side walls, such that only the top surface of the sample remained exposed. This configuration allowed the bottom and lateral surfaces to be isolated, enabling only the upper surface of the sealer to be subjected to analysis.

For radiopacity assessment (2.4.2), molds with dimensions of 10 mm in diameter and 1 mm in height were used.

### 2.3 Surface and elemental analysis

Macroscopic inspection, including color and surface texture assessment, was conducted on the same samples prepared for the radiopacity test (see below). Images were captured using a digital single-lens reflex (DSLR) camera (Nikon, Tokyo, Japan).

Scanning electron microscopy (SEM) was performed on sealers after 28 d of setting in 100% humidity at 37°C (*n* = 3). Specimens were progressively polished under water with increasingly fine abrasive discs, mounted on carbon tape, and examined using a SEM device (TM4000Plus II, Hitachi, Tokyo, Japan) at an accelerating voltage of 5–15 kV. High-magnification micrographs were captured using both backscattered and secondary electron modes to evaluate surface morphology. Energy-dispersive x-ray spectroscopy (EDS) was used for elemental analysis. EDS was conducted at 15 kV and a working distance of 10 mm, with data acquired from both spot and rectangular scan areas of the sealer surface.

### 2.4 ISO tests (6876:2012)

#### 2.4.1 Setting time

Sealers were injected into PET molds (*n* = 3) and incubated at 37°C at 100% humidity (INCU-Line incubator, VWR International, Radnor, PA, USA) until final setting. The setting endpoint was evaluated using a Gilmore-type indenter (mass: 100.0 g; flat tip diameter: 2.0 mm). The sealers were considered set when the indenter, gently placed on the surface, no longer left a visible circular indentation.

#### 2.4.2 Radiopacity

Specimens (*n* = 3; 10 mm in diameter, 1 mm in height) were allowed to set in PET molds (37°C at 100% humidity) for a duration 50% longer than the manufacturer's specified setting time (t₀). The samples were positioned on a photo-stimulable phosphor plate (VistaScan image plate 4+, Dürr Dental, Bietigheim-Bissingen, Germany), adjacent to a calibrated aluminum step wedge with 1 mm increments. Radiographic imaging was performed using a fixed x-ray box unit (Irix CCX, Trophy, Marne La Vallée, France) under standardized conditions: exposure time of 0.50 s, 10 mA, tube voltage of 65  ±  5  kV, and a source-to-film distance of 300 ± 10 mm. Radiographs were processed using the VistaScan Mini View system (Dürr Dental), and digital images were analyzed using ImageJ software (NIH, Bethesda, MD, USA), following the method described in detail elsewhere (17). Grey pixel values corresponding to the aluminum step wedge were used to generate a calibration curve, applying a logarithmic best-fit trend line for each image separately.

#### 2.4.3 Flowability

A volume of 0.05 mL of freshly mixed sealer was dispensed at the center of a glass plate. After 180  ±  5 s, a second glass plate (weight: 20 g) was gently placed on top, followed by a 100 g standard weight as described in the test instructions. Ten min after mixing, the largest and smallest diameters of the resulting disc were measured using a digital caliper. If the variation between measurements was less than 1 mm, the mean value was calculated. Otherwise, the test was repeated. Each material was tested in duplicate (*n* = 2), and the average flow value was recorded to the nearest 0.5 mm.

### 2.5 Microhardness

Microhardness testing was performed after 7 d of setting in 100% humidity at 37°C (*n* = 3) using a microhardness tester (Struers A/S, Rødovre, Denmark) equipped with a pyramidal, square-based diamond indenter. A load of up to 100 gf was applied for 5 s on the surface of each set specimen. At least two non-overlapping indentations, spaced 5 mm apart, were made on each sample. The Knoop Hardness Number (KHN) was recorded for each indentation.

### 2.6 pH assessment and calcium release

Triplicate specimens were immersed directly (without pre-setting) in 10 mL of phosphate-buffered saline (PBS) for 1 h, 1 d, 7 d, and 28 d. After each immersion, samples were transferred to 5 mL of Millipore water for 30 min. The pH and continued release of Ca²⁺ ions was assessed using a calibrated pH meter and atomic absorption spectroscopy (AAS), respectively. AAS was performed in a designated high-resolution continuum-source device (contrAA 300, Analytik Jena, Jena, Germany) equipped with a xenon short-arc lamp. Measurements were performed in a N_2_O/acetylene flame against a 6-step Ca standard (Merck, Darmstadt, Germany) dilution series from 1.0 to 0.0 ppm. Phosphate was masked using SrCl_2_ (Merck).

### 2.7 Aluminum release

For sealers identified by EDS to contain aluminum, analysis of cumulated aluminum ion release was performed using AAS (*n* = 3). Samples were immersed directly (without pre-setting) in 5 mL of Millipore water for 28 d prior to analysis. Measurements were performed as described above against a 7-step Al standard (Merck, Darmstadt, Germany) dilution series from 20 to 0 ppm Al. All the solutions, test and control, were spiked with hydrochloric acid (Merck) and cesium chloride-lanthanum chloride (Fluka, Buchs, Switzerland), so that they contained 1% HCl and 0.1% CsCl LaCl_3_ as an ionization buffer.

### 2.8 Data analysis

The present study was designed as a descriptive *in vitro* comparison of the physicochemical properties of currently marketed hydraulic silicate sealers. Several tests were performed according to ISO 6876:2012, which defines the execution and reporting of the respective measurements but does not require inferential statistical comparisons between materials. For example, the flowability test specifies the use of two samples and recording of the mean value. Therefore, considering the descriptive aim of the study and the small number of specimens used in some ISO-based tests, no inferential statistical analysis was performed. Data are presented as mean ± standard deviation where repeated measurements were performed, and no statistical significance between materials was claimed. Differences between materials were interpreted descriptively in relation to their composition and physicochemical behavior.

## 3 Results

### 3.1 Materials and their disclosed composition

The information regarding sealer components differed between the products under investigation (Table 1). Some companies revealed the weight distributions of the sealer components, while others did not. Seven sealers contained zirconia as their radiopacifier, while one product (NeoSealer Flo, Avalon Biomed) contained tantalum pentoxide. These elements were confirmed by EDS (see below). Dispersing agents were not disclosed in 5 of the 8 sealers, and cement components not specified in 4 (Table 1).

**Table 1:** Components of the sealers under investigation according to the safety data sheet and/or instructions for use.

Trade name	Cement components	Radiopacifier	Dispersing agent
BioRoot Flow	3CaO·SiO_2_	ZrO_2_	Polypropylene glycol
	CaCO_3_		Povidone
	SiO_2_		Acrylamide
			Sodium acryloyldimethyltaurate copolymer
			Isohexadecane
			Polysorbate
NeoSEALER Flo	3CaO·SiO_2_ (<25%)	Ta_2_O_5_ (<50%)	ND
	CaAl_2_O_4_ (<25%)		
	2CaO·SiO_2_ (<10%)		
	CaAl₄O₇ (<6%)		
	3CaO·Al_2_O_3_ (<1%)		
	CaSO_4_ (<1%)		
TotalFill BC Sealer	3CaO·SiO_2_ (<19%)	ZrO_2_ (<37%)	ND
	2CaO·SiO_2_ (<16%)		
	Ca(OH)_2_ (<14%)		
	CaSO_4_ (<9%)		
KometBioSeal	3CaO·SiO_2_ (<30%)	ZrO_2_ (<50%)	ND
	2CaO·SiO_2_ (<10%)		
	3CaO·Al_2_O_3_(<10%)		
One-Fil	Ca silicates (not specified, 40%)	ZrO_2_ (20%)	Polyethylene glycol 1500 & 1,3 Propanediol (20%)
C-Root SP	Ca phosphate (not specified, <10%)	ZrO_2_ (< 30%)	ND
	Sr silicate (not specified, <40%)		
	Ca(OH)_2_ (<5%)		
BC Universal Sealer	Ca aluminosilicate (not specified)	ZrO_2_ (<25%)	Polypropylene glycol (<7%)
	Ca Na phosphosilicate (not specified, <5%)	TiO_2_ (<7%)	
	CaSO_4_ (<5%)		
Bio-C SEALER ION+	Ca silicate (not specified)	ZrO_2_	ND
	Mg silicate (not specified)		
	Ca oxide		
	CaSO_4_		
	K₂SO₄		
	SiO_2_		

ND, not disclosed.

### 3.2 Surface and elemental analysis

**Figure 1:**
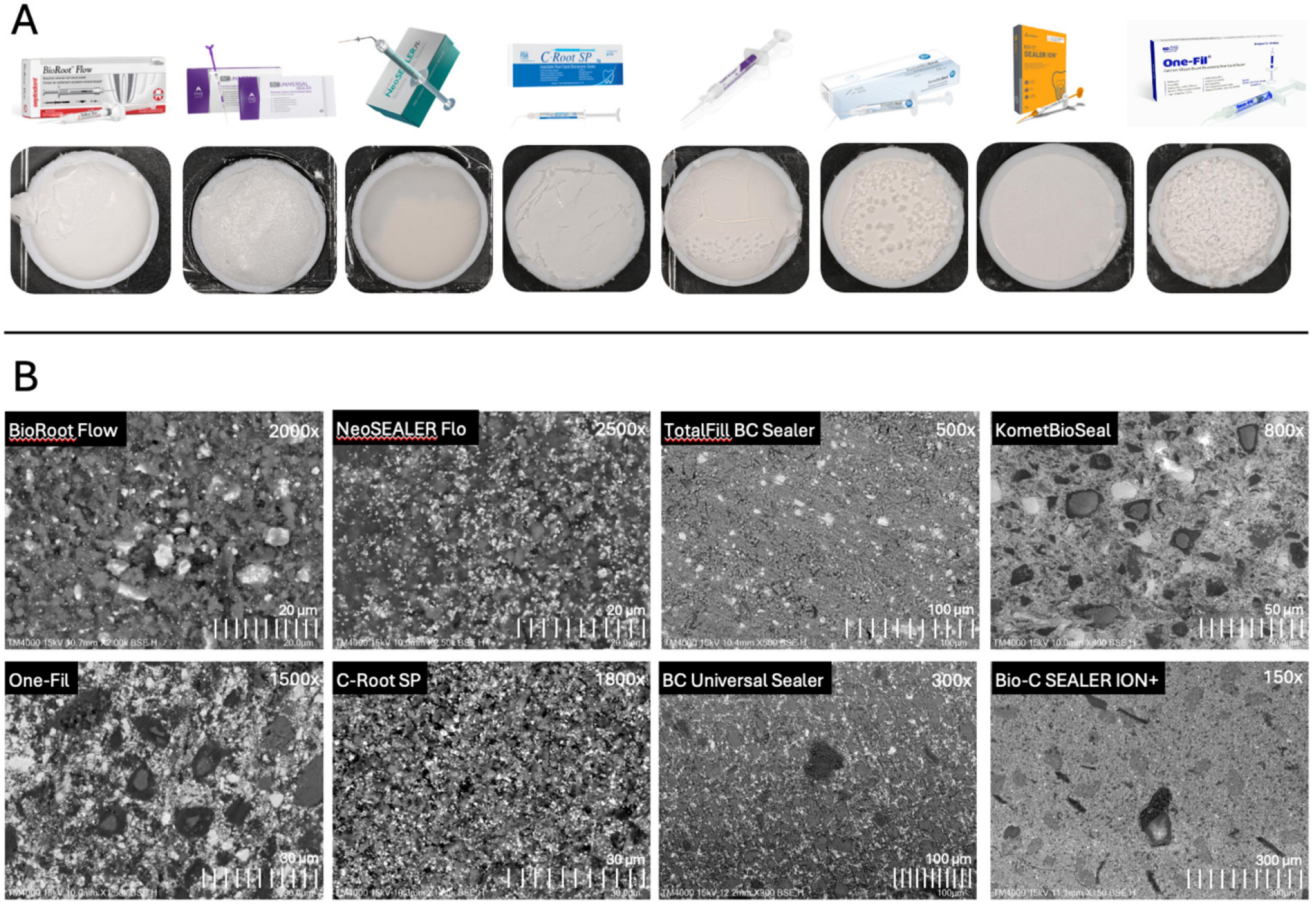
Representative microscopical images of the sealer surfaces showing different features including crystal-like formations **(Α)**. Representative backscatter scanning micrographs of the tested sealers (magnification 150×–2,500×).

The macroscopic and scanning electron microscopy (SEM) examination of the set sealers revealed notable differences in surface characteristics among the materials (Figure 1). BioRoot Flow (Septodont) and NeoSEALER Flo (Avalon Biomed) exhibited glossy surfaces, whereas BC Universal Sealer (Vericom) presented a more matte appearance. The remaining sealers displayed various crystalline formations on their surfaces, differing in size and morphology. KometBioSeal (Meta Biomed) and One-Fil (MEDICLUS), in particular, were entirely covered with apparent salt crystals.

SEM analysis also indicated structural differences among the sealers, including variations in the type, size, and distribution of the particles, which highlighted differences in composition and hydration process (Figure 1).

EDS analysis further revealed that all sealers contained Ca, silicon (Si), and oxygen (O). Al was detected in four products: NeoSEALER Flo (Avalon Biomed), KometBioSeal (Meta Biomed), One-Fil (MEDICLUS), and BC Universal Sealer (Vericom). Based on that observation and the information provided by the manufacturers, these four products can be classified as Portland cement-related sealers.

One sealer, Bio-C SEALER ION+ (Angelus), contained Mg silicate, and one, C-Root SP (C-Root Dental Medical), was a Sr silicate. These additional elements disclosed by the manufacturers (Table 1) were confirmed by EDS, as were titanium (Ti) in BC Universal Sealer (Vericom), and magnesium (Mg), potassium (K), and sulfur (S) in Bio-C SEALER ION + (Angelus). Phosphorus (P) was detected only in C-Root SP (C-Root Dental Medical) and TotalFill BC Sealer (Innovative BioCeramix), while chlorine (Cl) was detected in One-Fil (MEDICLUS) and KometBioSeal (Meta Biomed). The EDS spectra are presented in the Supplementary Material (Supplementary Image 1).

### 3.3 ISO tests

The ISO-requested flowability and radiopacity were met by all the sealers. All sealers reached the defined setting endpoint under the present experimental conditions, and none exceeded 72 h. However, none of the materials set within 30 min. The setting times deviated from those stated by the manufacturers (Supplementary Material; Supplementary Table 1). The ISO test results are presented in Table 2 and summarized below.

**Table 2:** Mean values ± standard deviations of setting time (min) and radiopacity (mm). Flow values were recorded to the nearest 0.5 mm.

Sealer	Setting time	Radiopacity	Flow
BioRoot Flow	1,797 ± 8	3.4 ± 0.1	26
NeoSEALER Flo	1,352 ± 8	5.4 ± 0.2	21
TotalFill BC Sealer	4,258 ± 14	6.1 ± 0.2	26
KometBioSeal	337 ± 8	7.4 ± 0.3	25
One-Fil	184 ± 7	7.4 ± 0.3	25
C-Root SP	605 ± 9	6.8 ± 0.3	22
BC Universal Sealer	122 ± 4	4.7 ± 0.1	24
Bio-C SEALER ION+	1,466 ± 9	5.5 ± 0.3	28

#### 3.3.1 Setting time

Setting time ranged from 122 ± 4 min for BC Universal Sealer to 4,258 ± 14 min for TotalFill BC Sealer. The sealers containing Al tended to set quicker than their Al-free counterparts, with the exception of NeoSEALER Flo (Avalon Biomed), which exhibited an intermediate setting time.

#### 3.3.2 Radiopacity

Radiopacity ranged from 3.4 ± 0.1 for BioRoot Flow to 7.4 ± 0.3 mm Al for One-Fil and KometBioSeal.

#### 3.3.3 Flowability

Flow values ranged from 21 mm for NeoSEALER Flo to 28 mm for Bio-C SEALER ION+. BioRoot Flow and TotalFill BC Sealer showed flow values of 26 mm, KometBioSeal and One-Fil 25 mm, BC Universal Sealer 24 mm, and C-Root SP 22 mm.

### 3.4 Microhardness

**Figure 2:**
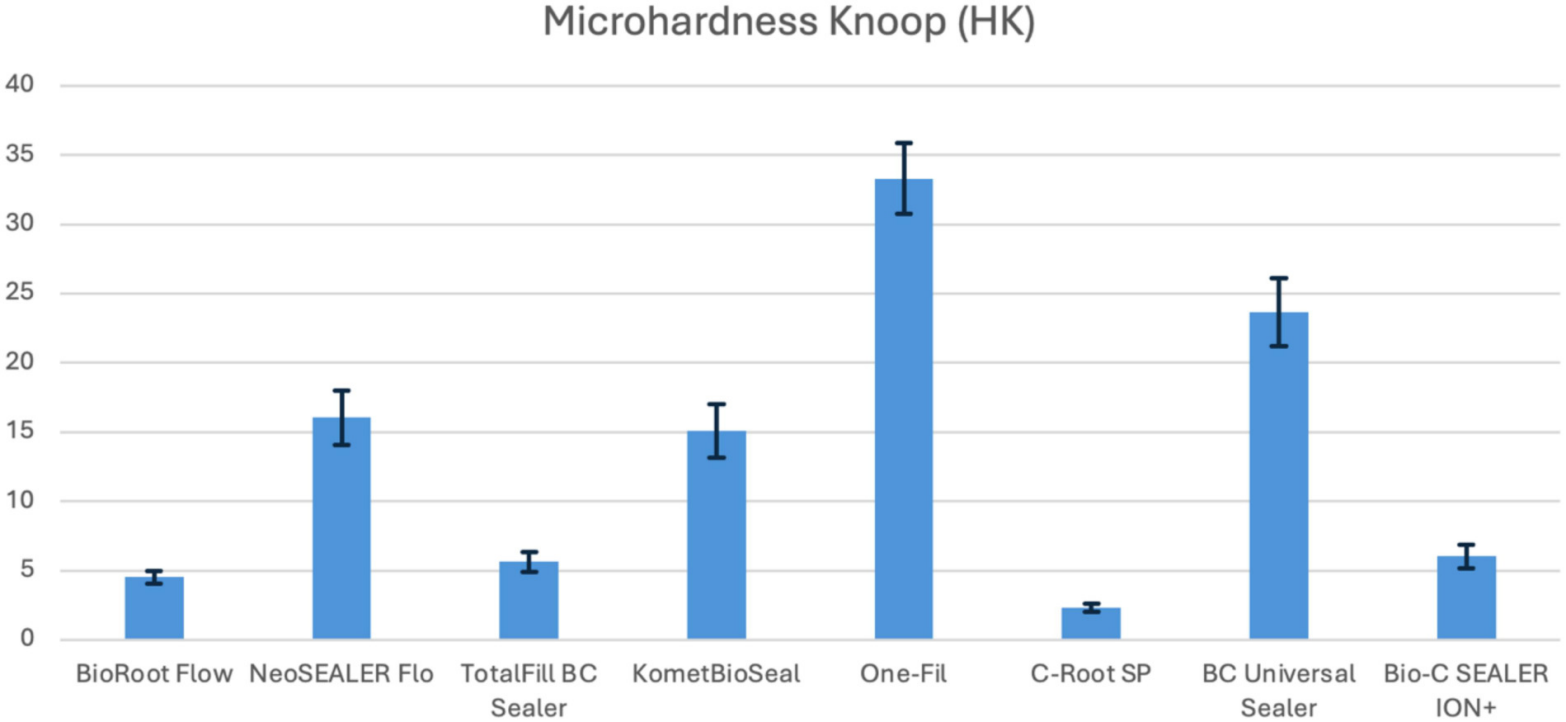
Mean and standard deviation of surface microhardness (Knoop Microhardness) of test materials after 7 d of setting. Note that the Portland cement-related sealers NeoSEALER Flo (Avalon Biomed), KometBioSeal (Meta Biomed), One-Fil (MEDICLUS), and BC Universal Sealer (Vericom) set harder than their Al-free counterparts.

Microhardness after setting differed considerably between the sealers under investigation. One product (C-Root SP, C-Root Dental Medical) exhibited a microhardness below 5 HK, whilst One-Fil (MEDICLUS) reached over 30 HK (Figure 2).

### 3.5 pH and leachates

**Figure 3:**
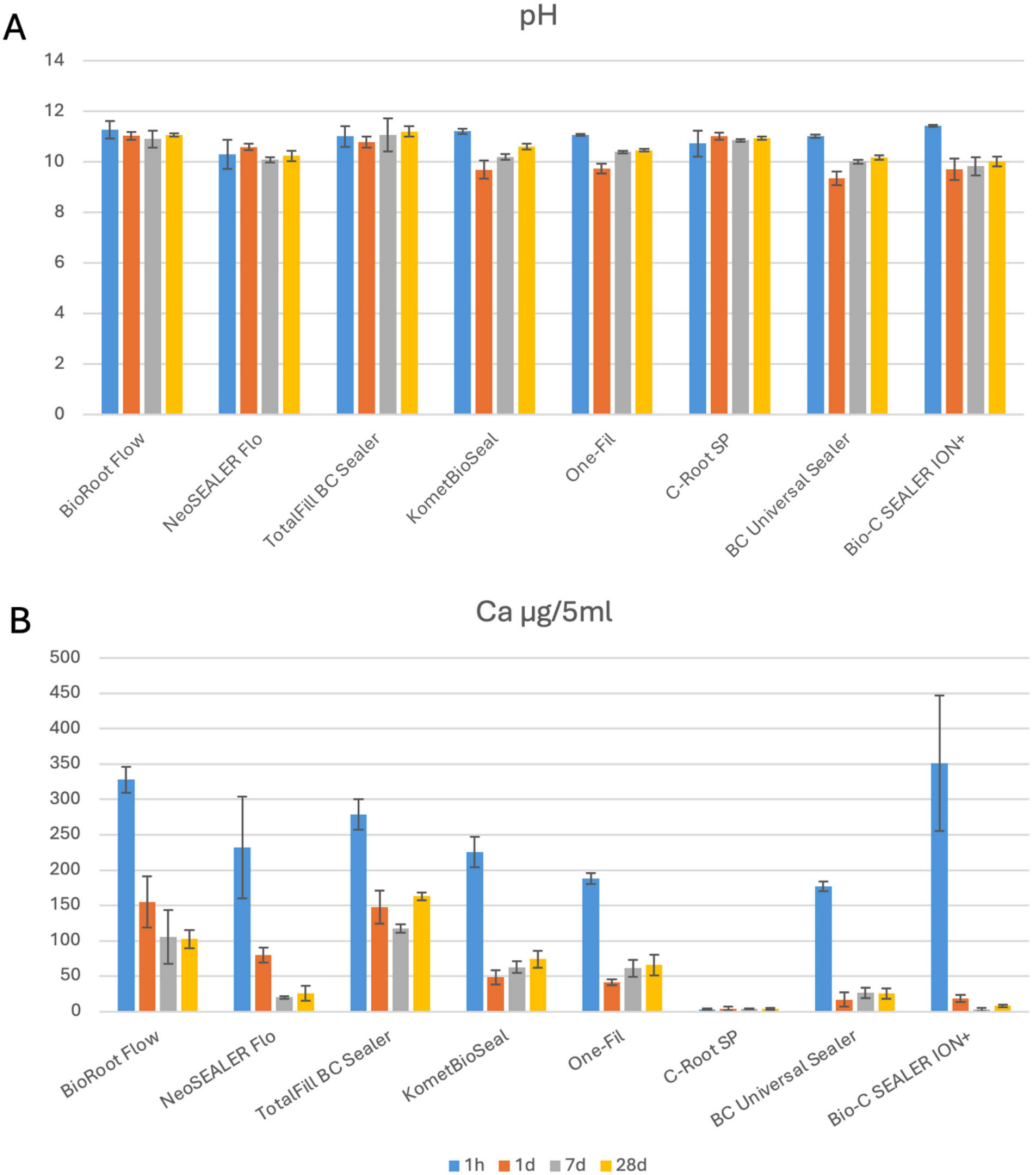
Mean and standard deviation of pH values **(A)** and Ca release **(B)** of the leachates of 1 h, 1-, 7- and 28-d set sealers.

All sealers maintained an alkaline pH induction throughout the observation period, with only minor fluctuations (Figure 3A). Peak Ca release was observed after 1 h of immersion in PBS, followed by a gradual decline over time. BioRoot Flow (Septodont) and TotalFill BC Sealer (Innovative BioCeramix) exhibited the highest levels of Ca release, whereas C-Root SP (C-Root Dental Medical) did not leach any Ca (Figure 3B).

**Figure 4:**
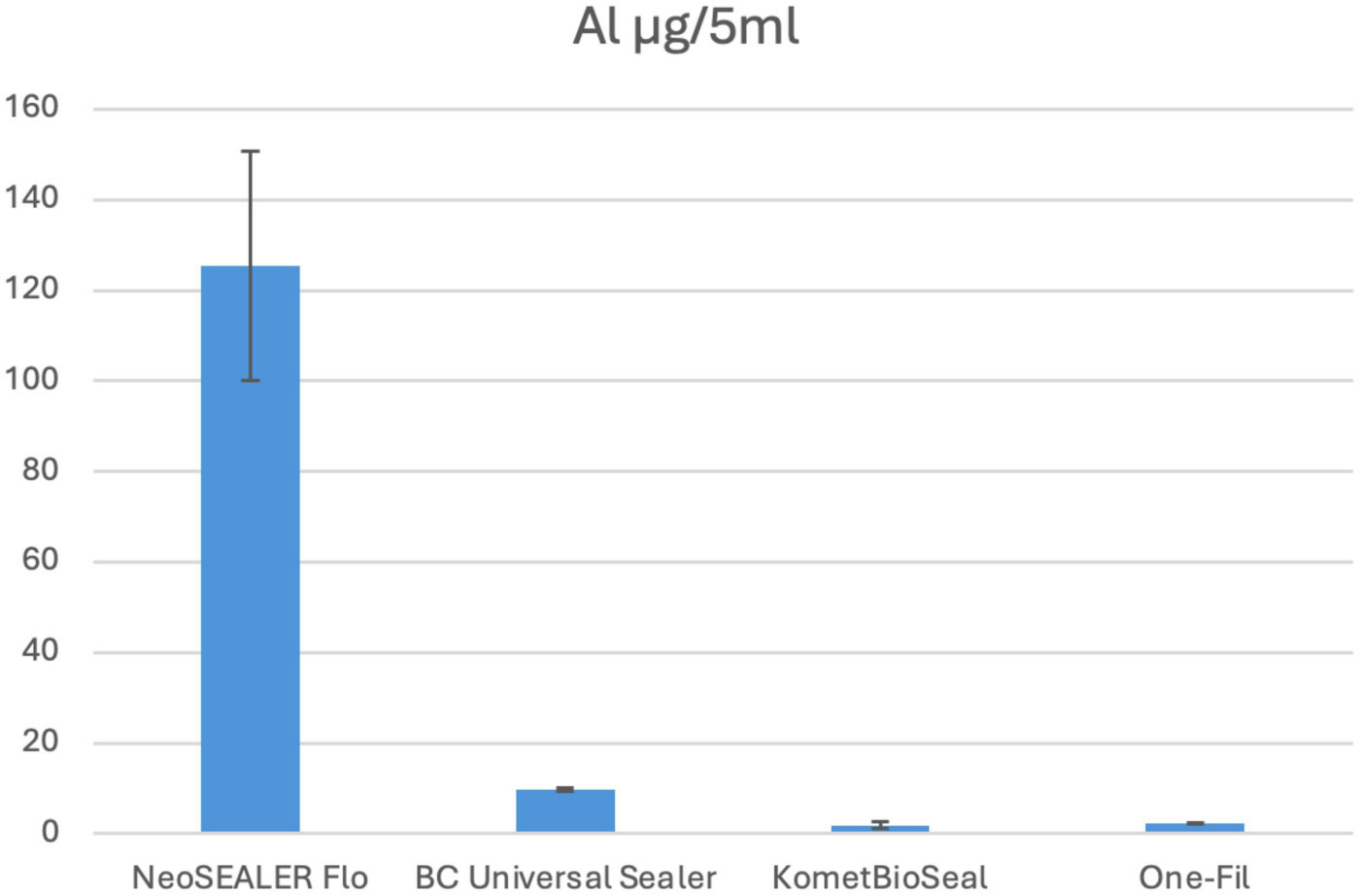
Mean and standard deviation of Al release of 28 d leachates of NeoSEALER Flo, BC Universal Sealer, KometBioSeal and One-Fil.

NeoSEALER Flo (Avalon Biomed) demonstrated the highest cumulated Al release after 28 d by far. In contrast, Al release from BC Universal Sealer (Vericom) was low, and that of KometBioSeal (Meta Biomed) and One-Fil (MEDICLUS) remained below the detection limit (Figure 4).

## 4 Discussion

The current study revealed considerable difference in the composition as well as the physico-chemical properties of currently marketed pre-mixed hydraulic silicate sealers. Apart from inducing a high pH in an aqueous environment and requiring an external water source to set, the sealers under investigation demonstrated differences in material composition and properties.

This study is limited by the fact that it was a pure bench-top investigation. No direct clinical conclusions can be drawn from these results. The true clinical risk or benefit of such materials can usually only be captured after several years in clinical use (18). The foundational rationale for this study was that currently marketed pre-mixed hydraulic silicate root canal sealers are often grouped under the same broad “bioceramic” category, although they may differ substantially in composition and physicochemical behavior. This distinction is clinically relevant because material selection is frequently influenced by general material-class claims rather than by product-specific data. Differences in physicochemical properties may influence handling, early material stability, and its interaction with dentin and periapical tissues. The present descriptive comparison therefore provides product-specific information to support a more differentiated interpretation of this material group and the data presented here may provide a valuable source of basic information on the materials under investigation.

The specifications provided regarding the components of the sealers under investigation differed greatly between manufacturers (Table 1). Only 3 manufacturers provided information regarding matrix components, while 5 did not (Table 1), which could cause uncertainty for dentists with patients who suffer from rare allergies, for example to dispersants such as polyethylene glycol (PEG) from root canal materials (16). Henceforth, all the components of a medical device need to be listed in the instructions for use (MDR, Annex I, Section 23). It should be cautioned, however, that the MDR is still not implemented fully (19), and not all the materials under investigation were MDR certified.

Why the composition of the sealers under investigation differed to the extent it did remains speculative. However, it can be assumed that patents, price of components, as well as some targeted features play a role in this. One material under investigation, Bio-C SEALER ION + (Angelus) contained a unique cement component in the form of åkermanite (Angelus, personal communication). The Mg contained in the åkermanite is believed to add to the bioactivity of this calcium silicate-based ceramic material (20), which is used in orthopedic applications (21). Whether this component is useful or makes a difference in endodontics remains to be elucidated.

In the present study, we examined various physico-chemical properties (plus hardness) of eight different premixed calcium silicate-based sealers. To the best of our knowledge, direct comparisons with the existing literature are limited, as many of the evaluated properties were tested for the first time for specific sealers. For instance, certain materials such as C-Root SP (C-Root Dental Medical) and Bio-C SEALER ION + (Angelus) have not been previously assessed for some of the tested parameters. Elemental analysis (EDX) results in our study were in agreement with previous findings (22–24). Regarding setting time, we observed significant variability among published studies, and no available data were found for BC Universal Sealer. Only KometBioSeal (Meta Biomed) and One-Fil (MEDICLUS) showed setting times comparable to those reported in previous studies (25, 26). The wide range of setting times should be interpreted in relation to the manufacturer-stated values and the ISO 6876:2012 framework. In the present study, all sealers reached the defined setting endpoint within 72 h, and no material was considered to have failed to set. However, none of the tested materials set within 30 min, and several sealers showed setting times outside the ISO/standard setting values or ranges stated by the manufacturers (Supplementary Material).

The wide variation in setting time across studies suggests that this property may be strongly influenced by methodology, environmental factors such as storage conditions and air exposure, highlighting the sensitivity of the materials under investigation to their surrounding environment (27). In terms of flow, our results for BioRoot Flow (Septodont), TotalFill BC Sealer (Innovative BioCeramix), NeoSEALER Flo (Avalon Biomed), KometBioSeal (Meta Biomed), and BC Universal Sealer (Vericom) aligned with those reported in several previous studies (13, 22, 25, 28, 29). However, some studies reported greater variability in flow values, possibly due to differences in methodology or sample handling. For radiopacity, our values were consistent with those reported for TotalFill BC Sealer, NeoSEALER Flo, KometBioSeal, One-Fil, and BC Universal Sealer in previous publications (13, 25, 28, 30).

All tested sealers maintained an alkaline pH, a finding that is consistent with the literature (22, 31). However, calcium ion release could not be directly compared with existing data due to differences in experimental design. Our method involved transferring the samples into fresh ultrapure water at specific intervals, allowing us to evaluate the continued pH induction and release of calcium ions over time, i.e., *capacity* (32). In contrast, other studies measured cumulative ion release within a single buffer solution (33), which may not accurately reflect the dynamic nature of ion exchange and precipitation at a certain time point. Finally, a low calcium ion release could also mean that most of the calcium ions are already trapped on the sealer surface as hydroxyapatite.

The bioactivity of hydraulic calcium silicate materials has been related to their Ca release (34). Therefore, continued release of Ca may be a goal of the manufacturers to advertise their product. TotalFill BC Sealer (Innovative BioCeramix Inc) is the product with the longest clinical track record among the tested materials. It showed a high and sustained Ca release in this and former studies (13). A sustained release of Ca was also observed with BioRoot Flow. This can be seen as a positive feature in terms of bioactivity. However, the sustained release over time together with the low hardness of these materials (Figure 2) can also be viewed as a sign that these synthetic CaSi materials do not set completely, which could contradict the Grossman concept of using inert materials for the hermetic seal of root canal systems (14). They may, however, provide a chemical seal against re-infection of the root canal system, which could be a topic for future investigations. On the other side of the spectrum, the Sr silicate sealer C-Root SP (C-Root Dental Medical) did not release any Ca. Sr release was not measured in this study, but it may be assumed that it occurred. Sr, a divalent cation, has similar biological properties as Ca (35). To our knowledge, microhardness has not been evaluated previously for these specific sealers. However, compressive strength has been reported (22). Microhardness is a multifactorial property that can serve as an indicator of material setting and surface integrity under different environmental conditions (36). Lower microhardness values have been associated with reduced bond strength to dentin and decreased sealing ability (37). In the present dataset, aluminum-containing sealers tended to show higher surface microhardness than aluminum-free silicate sealers. This observation should be interpreted cautiously, because surface microhardness is a multifactorial property and may be influenced not only by aluminum-containing phases, but also by cement chemistry and testing conditions.

One aspect that has been discussed with some controversy is the leaching of Al from aluminates in medical devices (11, 38), also with regard to the ever-increasing health awareness of patients. Our data confirm a published report on Al leaching from BC Universal Sealer (Vericom) (13). However, the Al leaching from NeoSEALER Flo (Avalon Biomed) exceeded those levels by far. This may not come as a surprise, because that material contains more than 50 wt% aluminates (Table 1). A recent cohort study found no correlation between Al dosage from vaccines received before age 2 and chronic diseases in children (39). The median dosage was 3 mg, which is one order of magnitude higher than the release reported here, observed with 0.5 g of sealer, i.e., more than is used per root canal, at maximum disposure. On the other hand, and given the ever-increasing health awareness of patients, there may still be a caveat with aluminosilicate root canal sealers. The biological relevance of this observation should be interpreted with caution. The present model represents an *in vitro* leaching scenario and does not directly reproduce clinical exposure. In clinical use, root canal sealers are generally confined within the root canal space and are surrounded by dentin and gutta-percha. Therefore, the amount of material directly exposed to periapical tissues or systemic circulation is expected to be limited. Under such conditions, the Al release observed here is unlikely to result in relevant systemic exposure or systemic toxicity. Nevertheless, local exposure may become more relevant when sealer is extruded beyond the apical foramen or placed in contact with periapical tissues, for example in teeth with open apices, resorption defects, or perforations. In such situations, biological responses would depend not only on the total amount of Al released, but also on the chemical form of the released Al species, local pH, solubility of the material, tissue contact area, and duration of exposure. Furthermore, Al-containing phases may influence other physicochemical properties, including setting time, hardness, and solubility. This may partly explain why several Al-containing materials in the present study showed shorter setting times and higher microhardness values than Al-free formulations. Thus, the present findings should not be interpreted as evidence of direct clinical toxicity. Rather, they indicate that hydraulic silicate sealers differ substantially in chemical formulation and leaching behavior, even though they are often marketed under the same general category of “bioceramic” sealers. From a clinical perspective, Al release is mainly relevant for material selection, manufacturer transparency, and post-market surveillance, especially in situations where extrusion or prolonged tissue contact may occur.

Regarding the limitations of this study, it was performed under standardized *in vitro* conditions and therefore cannot fully reproduce the clinical environment of the root canal system. The specimen geometry, exposed surface area, storage medium, and fluid volume were standardized to allow comparison among materials, but they do not reflect the complex interaction between sealer, dentin, residual moisture, irrigants, gutta-percha, and periapical tissues. The use of PET molds with enclosed bottom and side walls allowed standardized exposure of all materials, but also represents a simplified geometry, as only the upper specimen surface was exposed. The present study evaluated selected physicochemical properties and elemental release but did not assess biological properties. Therefore, the findings should be interpreted as product-specific laboratory data rather than direct predictors of clinical performance. Further studies addressing cytocompatibility, inflammatory responses, and clinically relevant exposure models are needed to determine whether the differences observed *in vitro* have biological significance.

## 5 Conclusions

The disclosing of product components was found to vary considerably between the different manufacturers of the sealers under investigation. Within the limitations of this descriptive *in vitro* study, the investigated pre-mixed hydraulic silicate root canal sealers showed product-dependent differences in composition, setting behavior, surface microhardness, calcium release, and aluminum leaching. All tested materials fulfilled the ISO 6876:2012 requirements for radiopacity and flowability under the present experimental conditions. The broad range of setting times and surface microhardness values indicated marked differences in material maturation and surface properties. Calcium release varied between materials. Among the Portland cement-related sealers, cumulative aluminum release was mainly observed in NeoSEALER Flo. These findings indicate that materials marketed under the umbrella term “bioceramic sealers” should not be regarded as a homogeneous material class, and their composition should be revealed more accurately by some manufacturers. Further clinical studies, biological investigations, and post-market surveillance are needed to determine which compositional and physicochemical features are clinically beneficial and which may be undesirable.

## Data Availability

The raw data supporting the conclusions of this article will be made available by the authors, without undue reservation.
