# Supplementary figures and images for: Physico-chemical analysis of eight pre-mixed hydraulic silicate sealers
Source: Front Dent Med. 2026 Jun 9;7:1851537. doi: 10.3389/fdmed.2026.1851537 (PMC13286903; doi:10.3389/fdmed.2026.1851537)

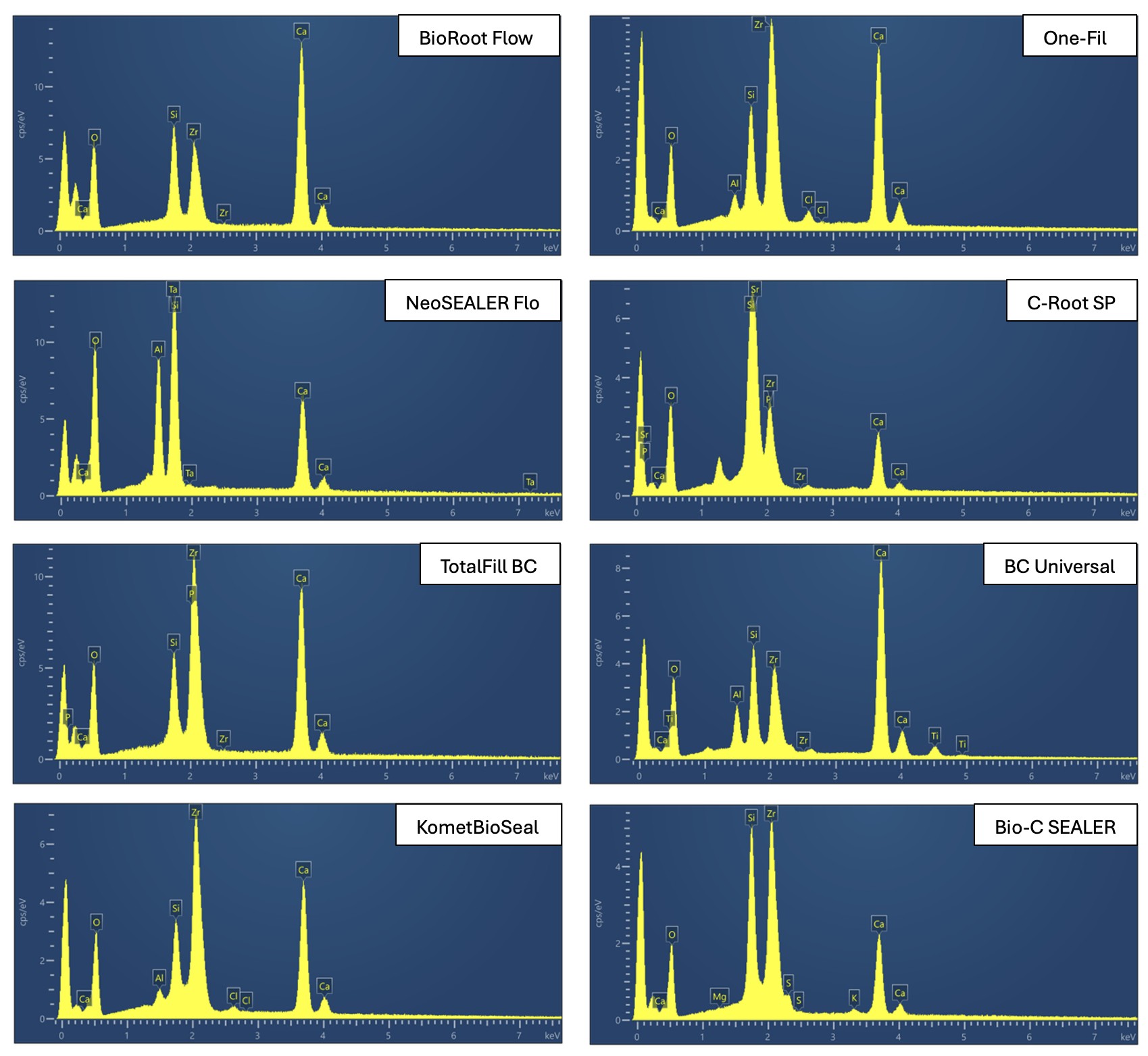

Supplement: Supplementary file 1 [file Image1.jpeg]
