# Supplementary material for: Physico-chemical analysis of eight pre-mixed hydraulic silicate sealers
Source: Front Dent Med. 2026 Jun 9;7:1851537. doi: 10.3389/fdmed.2026.1851537 (PMC13286903; doi:10.3389/fdmed.2026.1851537)
Supplement: Supplementary file 2 [file Table1.pdf]

Supplementary Table

Sealer	Manufacturer-stated setting time/ISO	Additional manufacturer note, not used for primary ISO comparison	Measured setting time in the present study	Comment
**BioRoot Flow**	45 min–8 h	—	1797 ± 8 min ≈ 30 h	Longer than the stated range; set within 72 h
**NeoSEALER Flo**	11 ± 1 h	—	1352 ± 8 min ≈ 22.5 h	Longer than the stated value/range; set within 72 h
**TotalFill BC Sealer**	4 h	Can be >10 h in very dry root canals	4258 ± 14 min ≈ 71 h	Substantially longer than the stated value; close to but below 72 h
**Komet BioSeal**	11 ± 1 h	—	337 ± 8 min ≈ 5.6 h	Shorter than the stated value/range; set within 72 h
**One-Fil**	30 min in 100% humidity	Within 3.5 h in root canal	184 ± 7 min ≈ 3.1 h	Exceeded the ISO 10% tolerance for a stated 30-min setting time; set within 72 h
**C-Root SP**	5–10 h	—	605 ± 9 min ≈ 10.1 h	At or slightly above the upper stated range; set within 72 h
**BC Universal Sealer**	25 min in 100% humidity	Can be >2.5 h in root canals / full setting around 150 min	122 ± 4 min ≈ 2.0 h	Exceeded the ISO 10% tolerance for a stated 25-min setting time; set within 72 h
**Bio-C SEALER ION+**	≤240 min / 30–240 min	Depends on canal humidity	1466 ± 9 min ≈ 24.4 h	Longer than the stated range; set within 72 h
